# Supplementary material for: Metabolomics Study on Pathogenic and Non-pathogenic E. coli with Closely Related Genomes with a Focus on Yersiniabactin and Its Known and Novel Derivatives
Source: Metabolites. 2020 May 28;10(6):221. doi: 10.3390/metabo10060221 (PMC7344775; doi:10.3390/metabo10060221)
Supplement: Supplementary file 1 [file metabolites-10-00221-s001.pdf]

metabolites

MDPI

Article

Metabolomics study on pathogenic and non-pathogenic *E. coli* with closely related genomes with focus on yersiniabactin and its known and novel derivatives

Mareike Schulz [1], Vasiliki Gaitanoglou [1], Olena Mantel [2], Yannick Hövelmann [1], Florian Hübner [1], Ulrich Dobrindt [2,*] and Hans-Ulrich Humpf [1,*]

[1] Institute of Food Chemistry, Westfälische Wilhelms-Universität Münster, Corrensstraße 45, 48149 Münster, Germany; m_sch065@uni-muenster.de (M.S.); vasilikigaitanoglou@yahoo.de (V.G.); yannick.hoevelmann@uni-muenster.de (Y.H); florian.huebner@uni-muenster.de (F.H.)

[2] Institute of Hygiene, Universitätsklinikum Münster, Germany, Mendelstraße 7, 48149 Münster, Germany; olena.mantel@ukmuenster.de (O.M)

* Correspondence: dobrindt@uni-muenster.de, Tel: +49-251-980-2875, Fax: +49-251-980-2868 (U.D.) humpf@uni-muenster.de, Tel: +49-251-83-33391, Fax: +49-251-83-33396 (H.-U.H.);

Supplementary Materials

Tables

Table S1. Comparison of intensities of the chosen features from the features list after data processing by MZmine 2.

Chosen discriminating features[1]	EcN MW replicate 1	EcN MW replicate 2	EcN MW replicate 3	*E. coli* 83972 MW replicate 1	*E. coli* 83972 MW replicate 2	*E. coli* 83972 MW replicate 3	*E. coli* CFT073 MW replicate 1	*E. coli* CFT073 MW replicate 2	*E. coli* CFT073 MW replicate 3	Blank MW 1
1, 482.1229, 13.25	2E+06	1E+06	2E+06	0E+00	0E+00	0E+00	0E+00	0E+00	0E+00	0E+00
2, 295.0565, 13.26	4E+06	3E+06	4E+06	0E+00	2E+04	5E+03	0E+00	0E+00	0E+00	0E+00
3, 482.1229, 14.21	5E+06	4E+06	5E+06	0E+00	0E+00	0E+00	0E+00	0E+00	0E+00	0E+00
4, 295.0565, 14.21	8E+06	7E+06	8E+06	0E+00	1E+05	0E+00	0E+00	0E+00	0E+00	0E+00
5, 535.0340, 11.00	2E+06	2E+06	2E+06	0E+00	6E+04	6E+03	0E+00	0E+00	0E+00	0E+00
6, 498.1178, 8.32	5E+05	4E+05	4E+05	5E+03	0E+00	0E+00	0E+00	0E+00	0E+00	0E+00
7, 311.0516, 8.31	1E+06	9E+05	1E+06	0E+00	6E+03	0E+00	0E+00	0E+00	0E+00	0E+00
8, 601.1270, 7.11	3E+06	3E+06	3E+06	0E+00	9E+04	7E+03	0E+00	4E+03	0E+00	0E+00
9, 414.0606, 7.12	1E+06	9E+05	1E+06	0E+00	7E+03	0E+00	0E+00	0E+00	0E+00	0E+00
10, 307.0201, 13.88	4E+05	3E+05	4E+05	0E+00	2E+04	0E+00	0E+00	0E+00	0E+00	0E+00
11, 365.0986, 14.42	2E+05	2E+05	2E+05	0E+00	0E+00	0E+00	0E+00	0E+00	0E+00	0E+00
12, 323.0550, 7.05	3E+06	2E+06	3E+06	1E+04	2E+05	5E+04	0E+00	0E+00	0E+00	0E+00
13, 279.0651, 7.05	6E+05	4E+05	5E+05	5E+03	2E+04	0E+00	0E+00	0E+00	0E+00	0E+00
14, 543.0369, 9.59	5E+06	4E+06	4E+06	2E+05	9E+05	2E+05	0E+00	0E+00	0E+00	0E+00
15, 355.9706, 9.58	6E+06	5E+06	5E+06	3E+05	1E+06	3E+05	0E+00	0E+00	0E+00	0E+00
16, 565.2345, 6.97	4E+06	4E+06	3E+06	2E+06	2E+06	2E+06	2E+07	2E+07	2E+07	0E+00

[1] No. of the feature, *m*/*z*, and retention time [min]

Table S2. Determined OD values at 600 nm of the culture samples of *E. coli* strains EcN, 83972 and CFT073. The cultivation was carried out for 6 h at 37 °C in minimum essential medium without shaking.

E. coli strain	Replicate	OD-value	pH
EcN	1	0.504	9.20
	2	0.451	9.21
	3	0.518	9.20
83972[1]	1	0.232	9.22
	2	0.222	9.22
	3	0.192	9.22
CFT073	1	0.515	9.17
	2	0.562	9.17
	3	0.468	9.18
Blank sample	1	-	9.11

[1]Visible formation of a biofilm at the bottom of the flask.

Table S3. Numerous novel derivatives of Ybt detected in the pre-concentrated culture supernatant of EcN during the isolation of ulbactin B. Characterization is based on their exact mass, isotope pattern and fragmentation behavior.

m/z **of [M+H]⁺ ion (measured)**	**Predicted molecular formula of [M+H]⁺**	***m/z*** **of [M+H]⁺ ion (calculated)**	**Different isomeric forms (abbreviation)**	
482.1239	$C_{21}H_{28}N_3O_4S_3^+$ (Δm 0.5 ppm)	482.1236	Isomer A_1[1] (Ybt-A_1)	Isomer A_2[1] (Ybt-A_2)
498.1188	$C_{21}H_{28}N_3O_5S_3^+$ (Δm 0.5 ppm)	498.1186	Isomer A[1] (**1**-A)	Isomer B (**1**-B)
601.1282	$C_{24}H_{33}N_4O_6S_4^+$ (Δm 0.8 ppm)	601.1277	Isomer A[1] (**2**-A)	Isomer B (**2**-B)
615.1433	$C_{25}H_{35}N_4O_6S_4^+$ (Δm -0.2 ppm)	615.1434	Isomer A (**3**-A)	Isomer B (**3**-B)
633.1003	$C_{24}H_{33}N_4O_6S_5^+$ (Δm -0.8 ppm)	633.0998	Isomer A (**4**-A)	Isomer B (**4**-B)
587.1127	$C_{23}H_{31}N_4O_6S_4^+$ (Δm 1.0 ppm)	587.1121	Isomer A (**5**-A)	Isomer B (**5**-B)
530.0911	$C_{21}H_{28}N_3O_5S_4^+$ (Δm 0.9 ppm)	530.0906	Isomer B (**6**-B)	Isomer A (**6**-A)
508.1391	$C_{23}H_{30}N_3O_4S_3^+$ (Δm -0.4 ppm)	508.1393	Three Isomers A (**7**-A_{1-3})	

[1]Features from the metabolomics study.

Table S4. Parameters for the chromatographic conditions for the metabolomics study based on the application of LC-HRMS.

**Column**	Nucleodur® C18 Gravity-SB column (150 x 2 mm, 3 µm) with a 4 x 2 mm Gravity SB guard column (Macherey-Nagel, Düren, Germany)		
**Gradient:**	Start	5% A	95% B
**A: ACN+0.1% FA**	0 - 1 min	5% A	95% B
**B: H_2O+0.1% FA**	up to 26 min	95% A	5% B
	26 – 28 min	95% A	5% B
	28 – 35 min	5% A	95% B
**Flow rate**	0.35 mL/min		
**Injection volume**	10 µL		
**Column temperature**	40 °C		
**Autosampler temperature**	4 °C		
**Additional detector**	DAD, 190 nm - 800 nm		

Table S5. Parameters for the LTQ Orbitrap XL™ mass spectrometer with heated electrospray ionization for the metabolomics study based on the application of LC-HRMS.

**Mass spectrometer**	LTQ Orbitrap XL™ equipped with HESI probe (Thermo Fisher Scientific, Dreieich, Germany)
**Source**	HESI
**Polarity**	positive
**Scan event**	1: Full scan, mass range: *m/z* 100-1000, resolution 30 000 (*m/z* 400), profile mode, AGC on, maximum fill time 25 ms
**Heater temperature**	350 °C
**Capillary temperature**	350 °C
**Sheath gas**	40 arbitrary units
**Aux gas**	20 arbitrary units
**Sweep gas**	10 arbitrary units
**Source voltage**	4 000 V
**Capillary voltage**	20 V
**Tube lens voltage**	125 V
**Diverter valve**	0-2 min: waste<br>2-35 min: MS
**Mass calibration**	External calibration (manufacturer's calibration mix) prior to running each sequence by manual injection using a syringe pump, mass accuracies < 1 ppm for calibrants

Table S6. Parameters of the different steps used for the data processing by MZmine 2 2.33 for the metabolomics study based on the application of LC-HRMS.

**Set sample parameters**			
**Peak detection**	Mass detection	Scans: RT 2-30 min	
		Mass detector: exact mass	
		Noise level: 1.2E4	
	Chromatogram builder	Scans: RT 2-30 min	
		Mass list: masses	
		Min time span (min): 0.05	
		Min height: 1.4E4	
		m/z tolerance: 5.0E-4 *m/z* or 3.0 ppm	
	Smoothing	Filter width: 5	
	Chromatogram deconvolution	Algorithm: local minimum search	Chromatographic threshold: 85%
			Search minimum in RT range (min): 0.10
			Minimum relative height: 0
			Minimum absolute height: 1.4E4
			Min ration of peak top/edge: 1.4
			Peak duration range (min): 0.05 - 1.00

Table S7. Parameters of the different steps used for the data processing by MZmine 2 2.33 for the metabolomics study based on the application of LC-HRMS (continued).

**Isotopes**	Isotopic peaks grouper	*m/z* tolerance:<br>5.0E-4 *m/z* or 3.0 ppm<br>RT tolerance:<br>0.1 absolute (min)<br>Monotonic shape<br>Maximum charge: 2<br>Representative isotope:<br>most intense
**Alignment**	Join aligner	*m/z* tolerance:<br>5.0E-4 m/z or 3.0 ppm<br>Weight for *m/z*: 2<br>RT tolerance:<br>0.3 absolute (min)<br>Weight for RT: 1
**Gap filling**	Peak finder	Intensity tolerance: 50%<br>*m/z* tolerance:<br>5.0E-4 *m/z* or 3.0 ppm<br>RT tolerance:<br>0.1 absolute (min)

Table S8. Changed parameters of the fragmentation experiments for the LTQ Orbitrap XL™ mass spectrometer with heated electrospray ionization for the metabolomics study based on the application of LC-HRMS.

**Scan event:**

**1**: Full scan, mass range: *m/z* 100-1000, resolution 30 000 (*m/z* 400), profile mode, AGC on, maximum fill time	**2**: mass list (**1** or **2**), CID (35% relative normalized collision energy, activation time 30 ms, isolation width 1.7 Da), resolution 7 500 (*m/z* 400), centroid mode, AGC on, maximum fill time 100 ms, dynamic exclusion enabled (repetition count 3, repetition duration 15 s, exclusion duration 90 s)	**3**: data-dependent fragmentation of most intense ion observed in scan 2, CID (35% relative normalized collision energy, activation time 30 ms, isolation width 1.7 Da), resolution 7 500 (*m/z* 400), centroid mode, AGC on, maximum fill time 100 ms, dynamic exclusion enabled (repetition count 3, repetition duration 15 s, exclusion duration 90 s)
**Mass list 1** (for MS2 and MS3): 307.02, 365.10, 482.12, 498.12, 530.09, 587.11, 601.13, 615.14, 633.10		
**Mass list 2** (ions after in source fragmentation, for MS3 and MS4): 293.04, 295.06, 311.05, 343.02, 414.06, 428.08, 446.03		

Table S9. Parameters for the chromatographic conditions for LC-MS/MS analysis.

**Column**	Nucleodur® C18 Gravity-SB column (10 x 2 mm, 3 µm) with a 4 x 2 mm Gravity SB guard column (Macherey-Nagel, Düren, Germany)		
**Gradient:** **A: ACN+0.1% FA** **B: H_2O+0.1% FA**	Start	10% A	90% B
	0 – 0.5 min	10% A	90% B
	0.5 – 5 min	30% A	70% B
	5 – 13 min	95%A	5% B
	13 – 15 min	95% A	5% B
	15 – 20 min	10% A	90% B
**Flow rate**	0.40 mL/min		
**Injection volume**	10 µL		
**Column temperature**	40 °C		
**Autosampler temperature**	4 °C		

Table S10. Parameters for QTRAP® 5500 with electrospray ionization for LC-MS/MS analysis.

**Mass spectrometer**	QTRAP® 5500 (Sciex, Darmstadt, Germany)	**Source**	ESI

**Scan type**	MRM		**Source Temperature**	500 °C
**Polarity**	Positive		**Source voltage**	5 500 V
**Quadrupole resolution**	Unit		**Curtain gas (CUR)**	25 psi
**Declustering potential (DP)**	100 V		**Nebulizer gas (GS1)**	35 psi
**Entrance potential (EP)**	10 V		**Heater gas (GS2)**	45 psi
**Collision Cell Exit Potential (CXP)**	11 V		**Diverter valve**	0-2 min: waste 2-20 min: MS
**Analyte ion $[M+H]^+$**	**Q1 [*m*/*z*]**	**Q3 [*m*/*z*]**	**Dwell time [ms]**	**Collision energy [V]**
$[Ybt+H]^+$	482.0	295.0	15	26
	482.0	190.0	15	49
$[Fe(III)\text{-}Ybt+H]^+$	535.0	348.0	15	22
	535.0	303.0	15	42
$[Cu(II)\text{-}Ybt+H]^+$	543.0	356.0	15	21
	543.0	294.0	15	43
$[Escherichelin+H]^+$	307.0	261.0	15	30
	307.0	203.0	15	30
$[Ulbactin\ B+H]^+$	365.0	212.0	15	42
	365.0	190.2	15	42
[(**1**-A)+H]$^+$	498.0	311.0	15	26
	498.0	293.0	15	26
[(**2**-A)+H]$^+$	601.0	414.0	15	26
	601.0	261.0	15	42

Table S11. Parameters for the chromatographic separation for the isolation of ulbactin B by preparative HPLC-UV.

**Column**	Nucleodur® Phenyl-Hexyl column (250 x 10 mm, 5 µm) with a 4 x 2 mm Phenyl-Hexyl guard column (Macherey-Nagel, Düren, Germany)		
**Gradient:**	Start	20% A	80% B
**A: ACN+0.1% FA**	0 – 0.5 min	20% A	80% B
**B: H_2O+0.1% FA**	0.5 – 20 min	95%A	5% B
	20 – 22 min	95% A	5% B
	22 – 29 min	20% A	80% B
**Flow rate**	4.5 mL/min		
**Injection volume**	250 µL		
**Column temperature**	40 °C		
**Detector**	DAD, 254 nm		

Figures

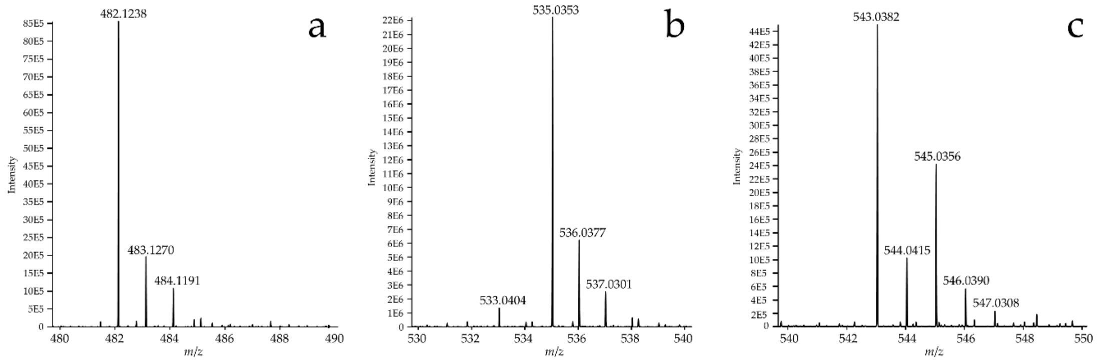

Figure S1. Isotope pattern of Ybt (a; feature No. *1 and *3, [M+H]⁺: $C_{21}H_{28}N_3O_4S_3^+$, calculated: *m/z* 482.1236, measured: *m/z* 482.1238, Δm 0.3 ppm), Fe(III)-Ybt (b; feature No. *5, [M+H]⁺: $C_{21}H_{25}FeN_3O_4S_3^+$, calculated: *m/z* 535.0351, measured: *m/z* 535.0353, Δm 0.4 ppm) and Cu(II)-Ybt (c; feature No. *14, [M+H]⁺: $C_{21}H_{26}CuN_3O_4S_3^+$, calculated: *m/z* 543.0376, measured: *m/z* 543.0382, Δm 1.1 ppm) by the application of LC-HRMS (LTQ Orbitrap XL™).

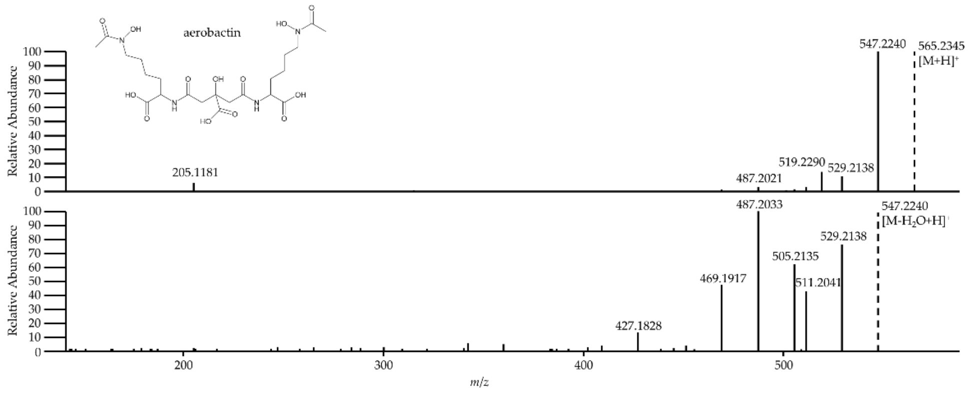

Figure S2. Fragmentation spectrum of aerobactin by the application of LC-MS/HRMS (LTQ Orbitrap XL™, CID, 35%). Spectra shown: MS^2 spectrum of the $[M+H]^+$ ion (*m/z* 565.2345) and MS^3 spectrum of the $[M-18+H]^+$ ion (*m/z* 547.2240).

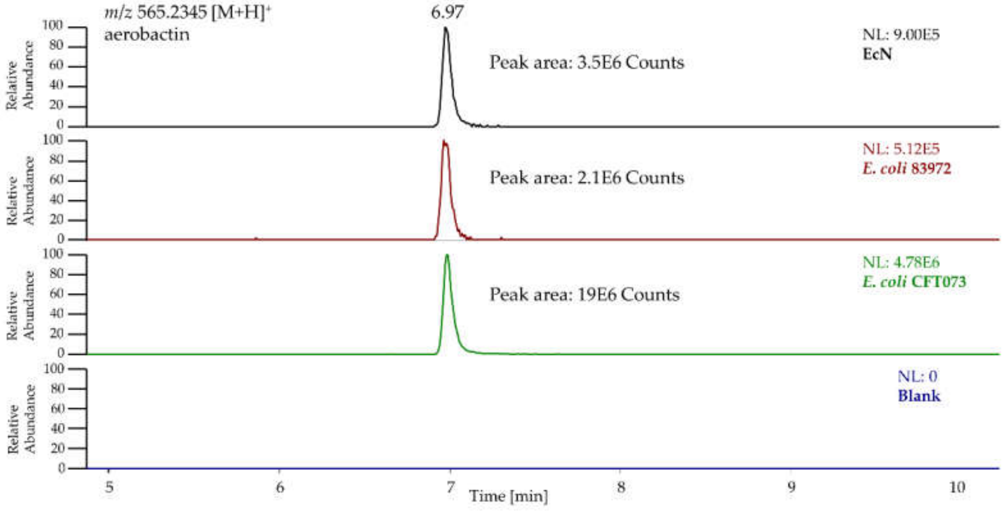

Figure S3. EIC (feature No. *16, $[M+H]^+$: $C_{22}H_{37}N_4O_{13}^+$, calculated: *m/z* 565.2352, measured: *m/z* 565.2345, Δm -1,3 ppm) of the culture supernatant from *E. coli* strains EcN, 83972 and CFT073 and the blank sample by the application of LC-HRMS (LTQ Orbitrap XL™).

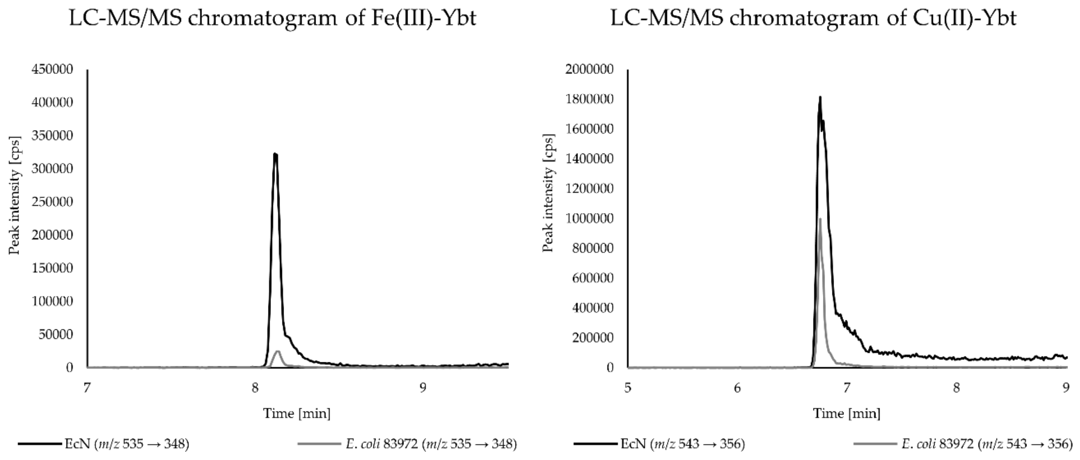

Figure S4. LC-MS/MS chromatogram (QTRAP® 5500) of the culture supernatant from *E. coli* strains EcN and 83972, displaying the two metal complexes of Ybt with the transition *m/z* 535 → 348 for Fe(III)-Ybt and *m/z* 543 → 356 for Cu(II)-Ybt.

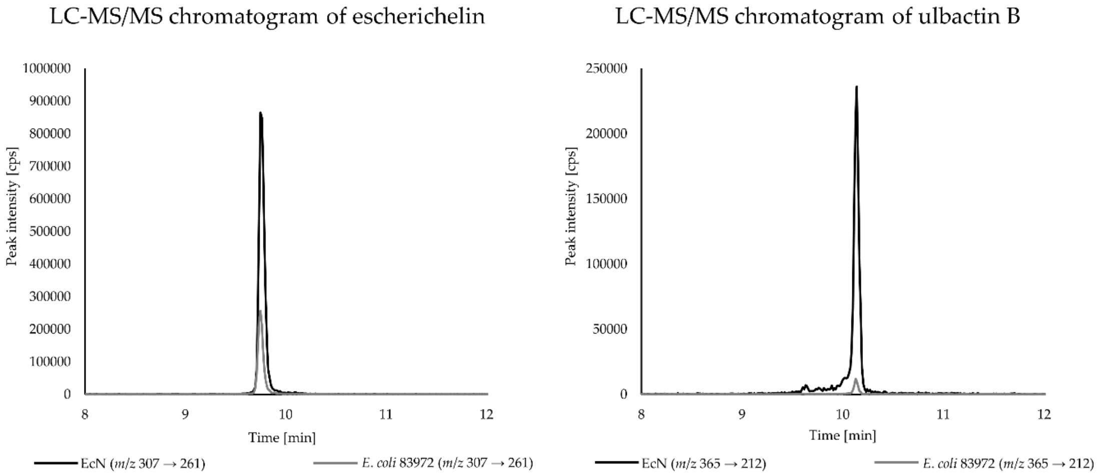

Figure S5. LC-MS/MS chromatogram (QTRAP® 5500) of the culture supernatant from *E. coli* strains EcN and 83972, displaying the known derivatives of Ybt with the transition *m/z* 307 → 261 for escherichelin and *m/z* 365 → 212 for ulbactin B.

LC-MS/MS chromatogram of the derivative **1**-A LC-MS/MS chromatogram of the derivative **2**-A

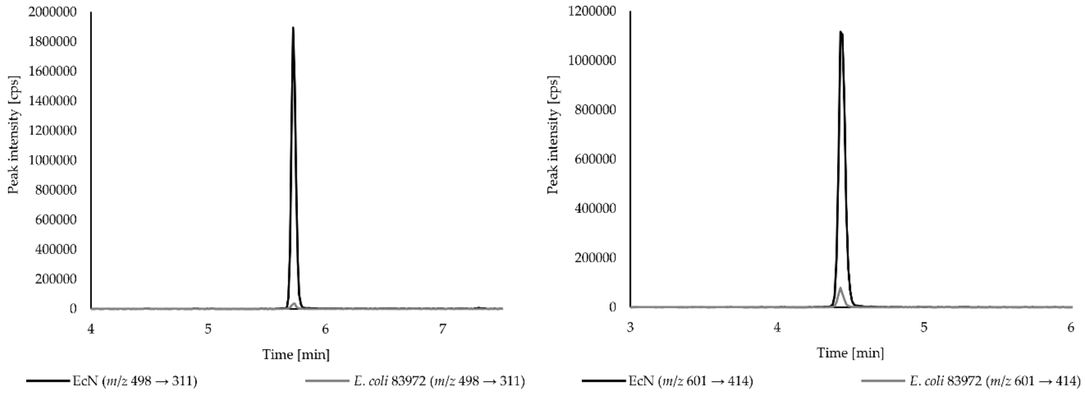

Figure S6. LC-MS/MS chromatogram (QTRAP® 5500) of the culture supernatant from *E. coli* strains EcN and 83972, displaying the novel Ybt derivatives with the transition *m*/*z* 498 → 311 for **1**-A and *m*/*z* 601 → 414 for **2**-A.

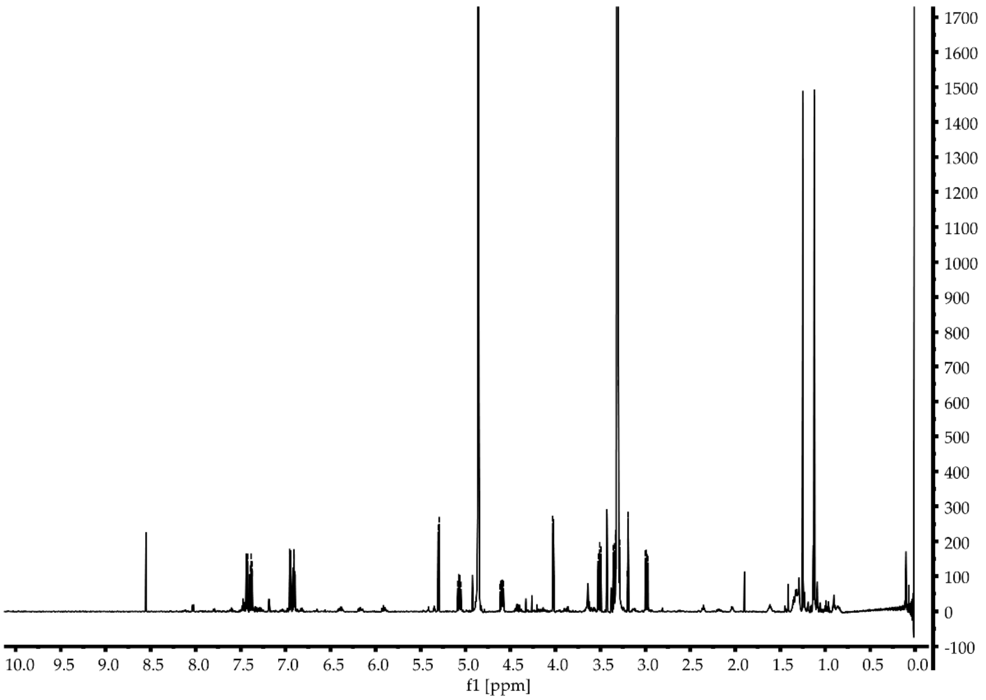

Figure S7. ^{1}H NMR spectrum (600 MHz) of 0.2 mg ulbactin B in MeOD isolated from culture supernatant of EcN for identification in the metabolomics study.

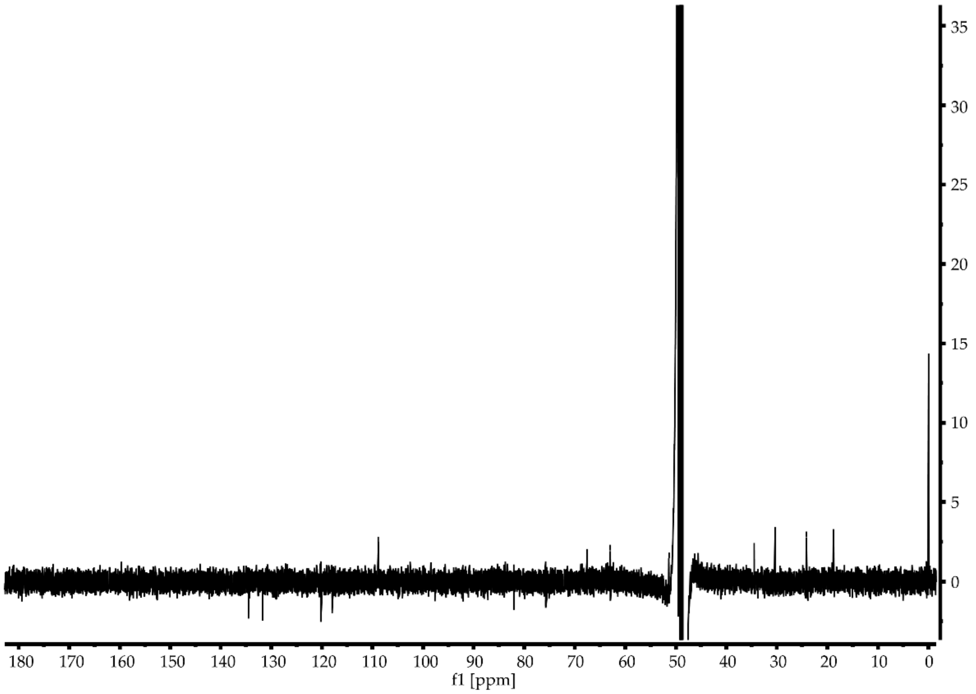

Figure S8. ^{13}C NMR spectrum of 0.2 mg ulbactin B in MeOD isolated from culture supernatant of EcN for identification in the metabolomics study.

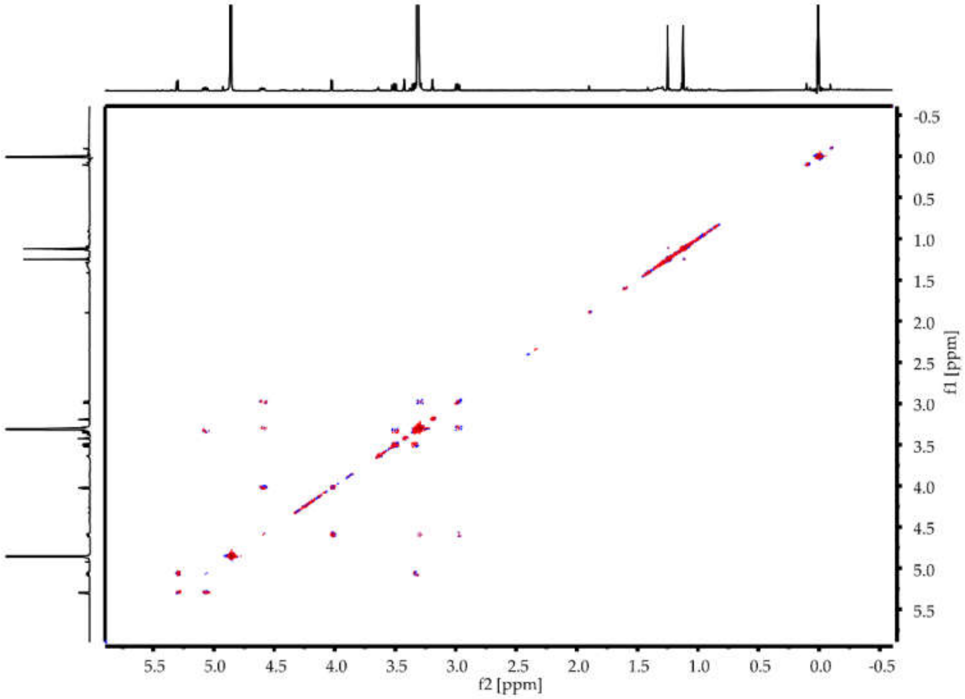

Figure S9. gCOSY NMR spectrum of 0.2 mg ulbactin B in MeOD isolated from culture supernatant of EcN for identification in the metabolomics study.

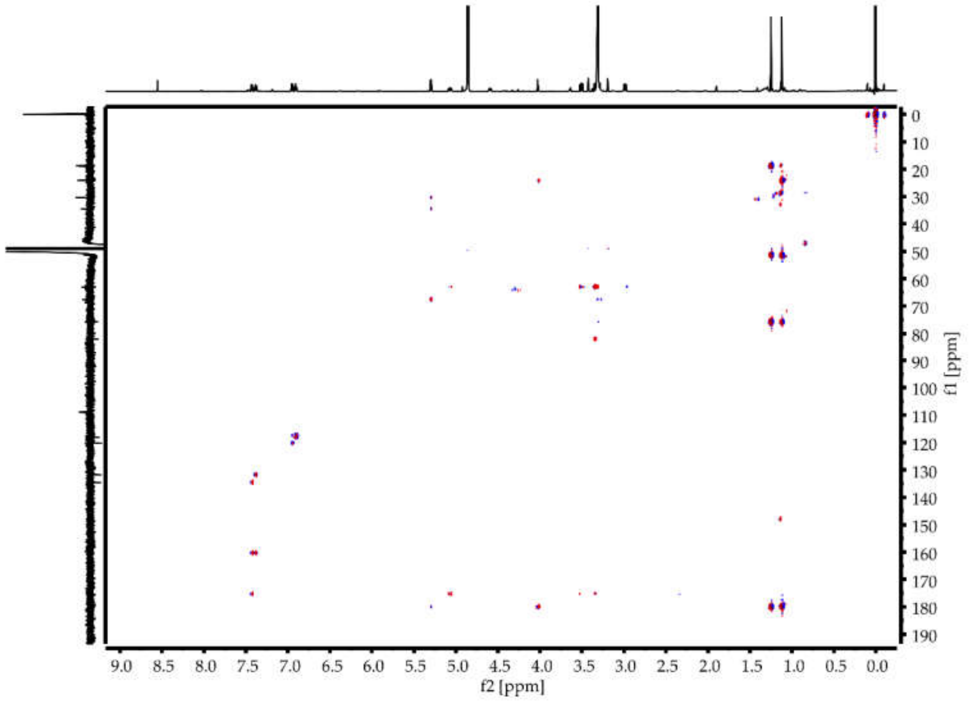

Figure S10. gHMBC NMR spectrum of 0.2 mg ulbactin B in MeOD isolated from culture supernatant of EcN for identification in the metabolomics study.

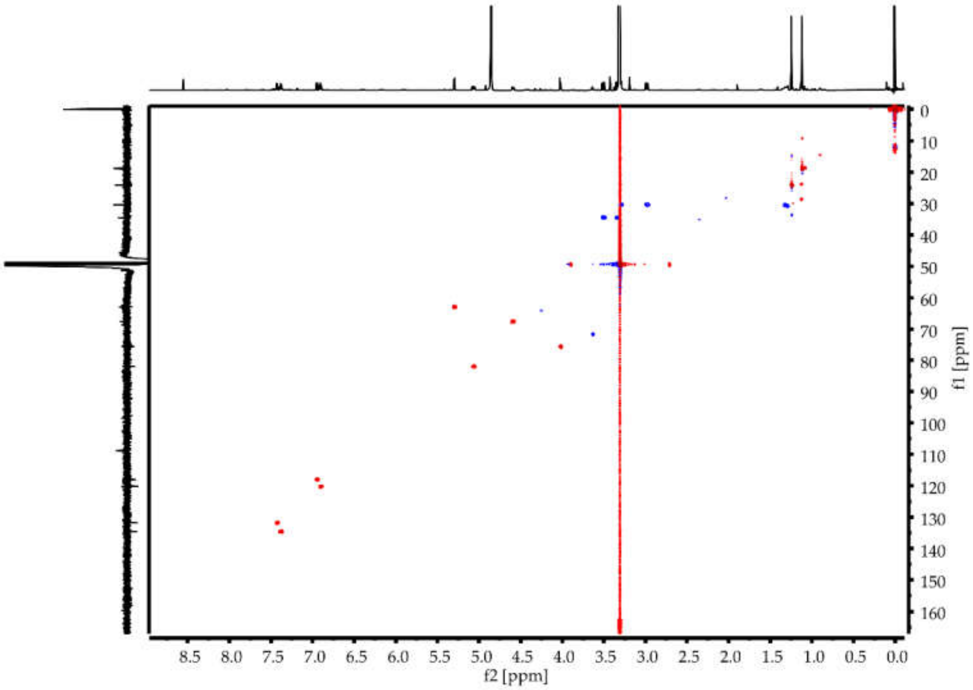

Figure S11. gHSQC NMR spectrum of 0.2 mg ulbactin B in MeOD isolated from culture supernatant of EcN for identification in the metabolomics study.

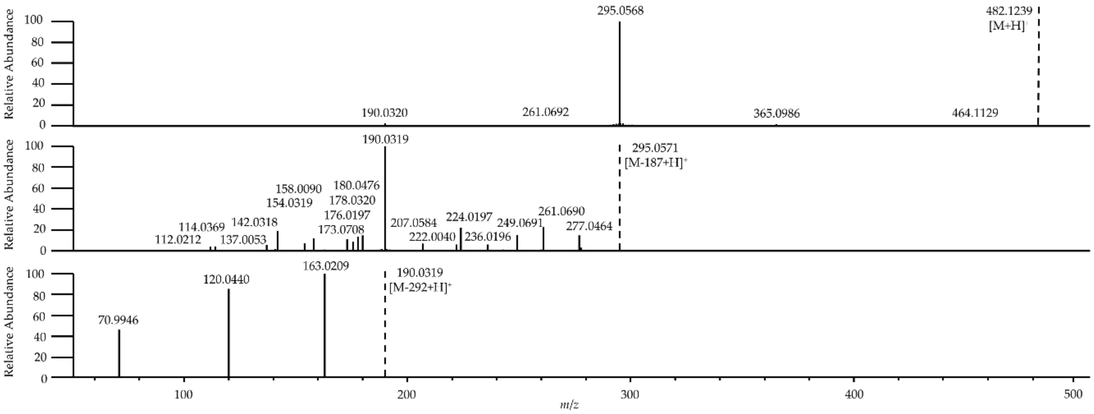

Figure S12. Fragmentation spectrum of isomer A1 and A2 of Ybt by the application of LC-MS/HRMS (LTQ Orbitrap XL™, CID, 35%). Spectra shown: MS² spectrum of the [M+H]⁺ ion (*m/z* 482.1239), MS³ spectrum of the [M-187+H]⁺ ion (*m/z* 295.0571) and MS⁴ spectrum of the [M-292+H]⁺ ion (*m/z* 190.0319).

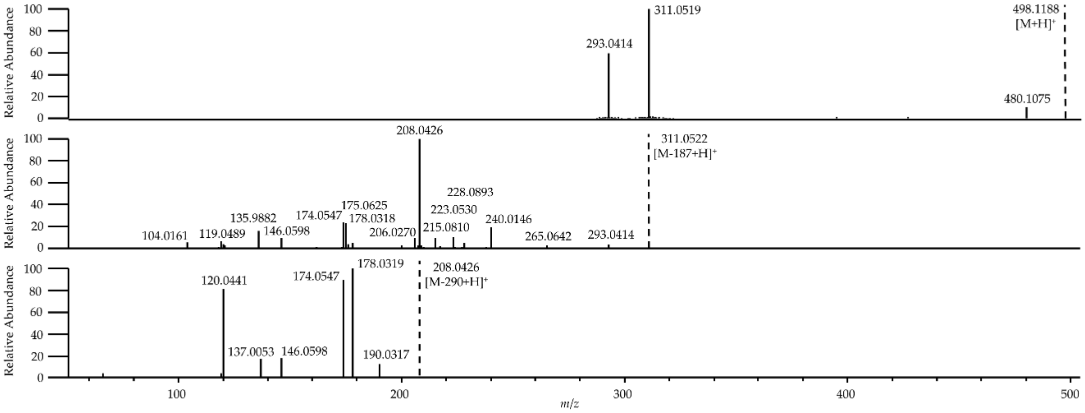

Figure S13. Fragmentation spectrum of the novel derivative **1**-A by the application of LC-MS/HRMS (LTQ Orbitrap XL™, CID, 35%). Spectra shown: MS² spectrum of the [M+H]⁺ ion (*m/z* 498.1188), MS³ spectrum of the [M-187+H]⁺ ion (*m/z* 311.0522) and MS⁴ spectrum of the [M-290+H]⁺ ion (*m/z* 208.0426).

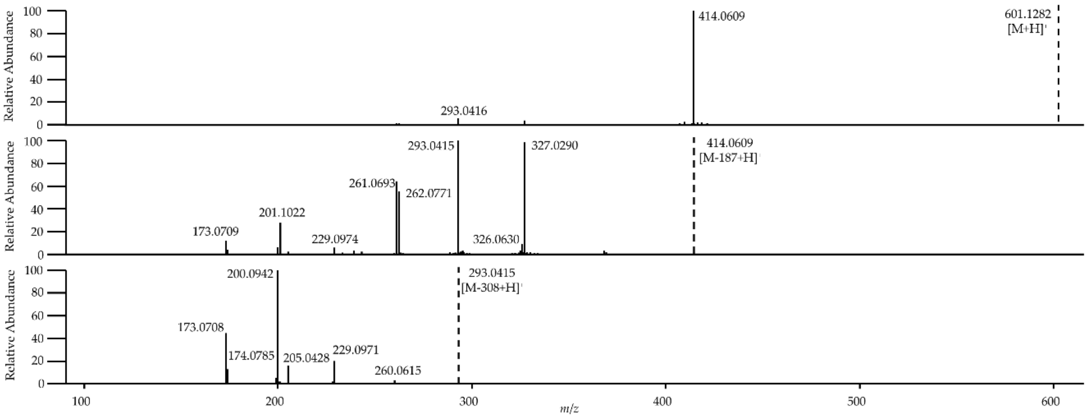

Figure S14. Fragmentation spectrum of the novel derivative **2**-A by the application of LC-MS/HRMS (LTQ Orbitrap XL™, CID, 35%). Spectra shown: MS^2 spectrum of the $[M+H]^+$ ion (*m/z* 601.1282), MS^3 spectrum of the $[M-187+H]^+$ ion (*m/z* 414.0609) and MS^4 spectrum of the $[M-308+H]^+$ ion (*m/z* 293.0415).

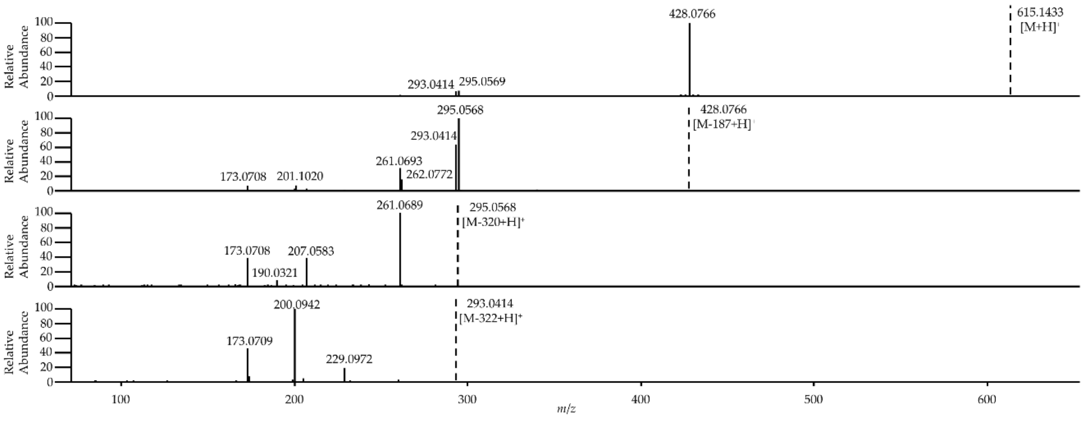

Figure S15. Fragmentation spectrum of the novel derivative **3**-A by the application of LC-MS/HRMS (LTQ Orbitrap XL™, CID, 35%). Spectra shown: MS^2 spectrum of the $[M+H]^+$ ion (*m/z* 615.1433), MS^3 spectrum of the $[M-187+H]^+$ ion (*m/z* 428.0766) and MS^4 spectrum of the $[M-320+H]^+$ ion (*m/z* 295.0568) and of the $[M-322+H]^+$ ion (*m/z* 293.0414).

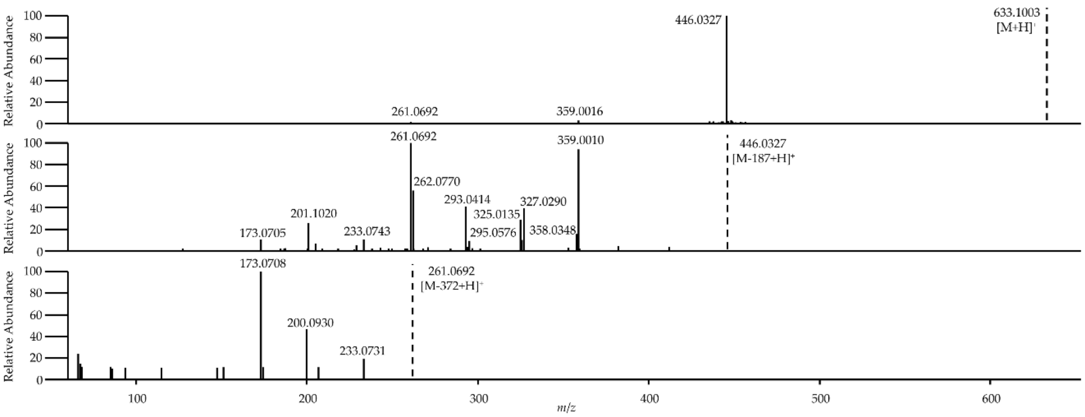

Figure S16. Fragmentation spectrum of the novel derivative **4**-A by the application of LC-MS/HRMS (LTQ Orbitrap XL™, CID, 35%). Spectra shown: MS^2 spectrum of the $[M+H]^+$ ion (*m/z* 633.1003), MS^3 spectrum of the $[M-187+H]^+$ ion (*m/z* 446.0327) and MS^4 spectrum of the $[M-372+H]^+$ ion (*m/z* 261.0692).

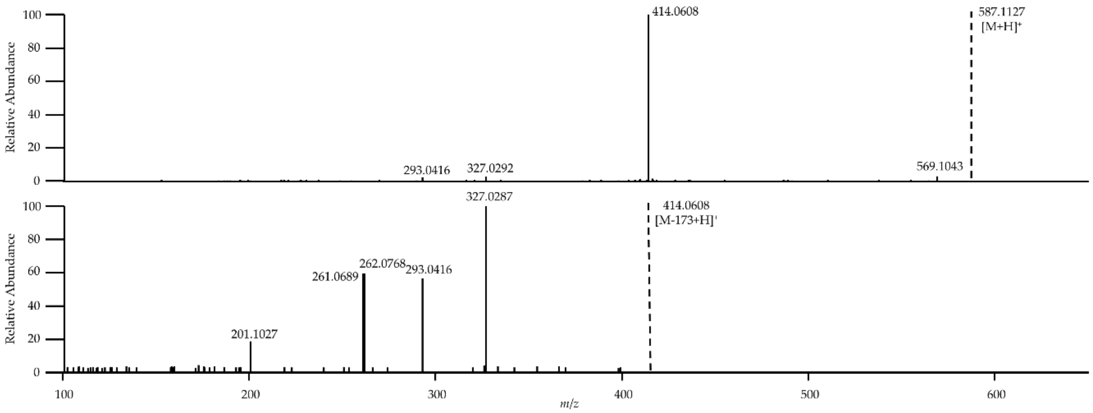

Figure S17. Fragmentation spectrum of the novel derivative **5**-A by the application of LC-MS/HRMS (LTQ Orbitrap XL™, CID, 35%). Spectra shown: MS^2 spectrum of the $[M+H]^+$ ion (*m/z* 587.1127) and MS^3 spectrum of the $[M-173+H]^+$ ion (*m/z* 414.0608).

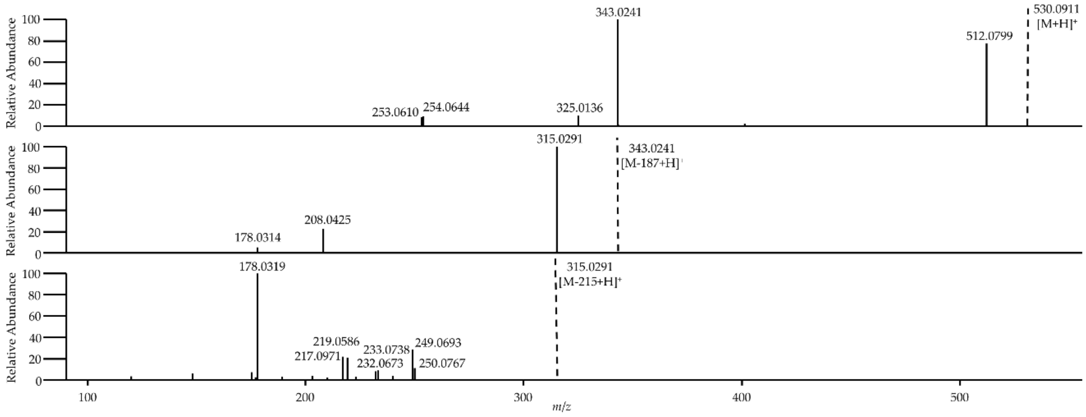

Figure S18. Fragmentation spectrum of the novel derivative **6**-A by the application of LC-MS/HRMS (LTQ Orbitrap XL™, CID, 35%). Spectra shown: MS^2 spectrum of the $[M+H]^+$ ion (*m/z* 530.0911), MS^3 spectrum of the $[M-187+H]^+$ ion (*m/z* 343.0241) and MS^4 spectrum of the $[M-215+H]^+$ ion (*m/z* 315.0291).

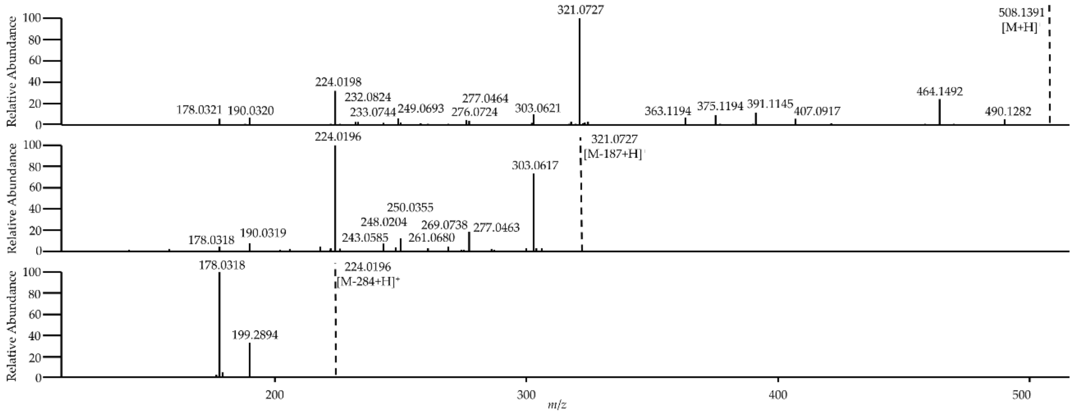

Figure S19. Fragmentation spectrum of the novel derivatives **7**-A1-3 by the application of LC-MS/HRMS (LTQ Orbitrap XL™, CID, 35%). Spectra shown: MS² spectrum of the [M+H]⁺ ion (*m/z* 508.1391), MS³ spectrum of the [M-187+H]⁺ ion (*m/z* 321.0727) and MS⁴ spectrum of the [M-284+H]⁺ ion (*m/z* 224.0196).

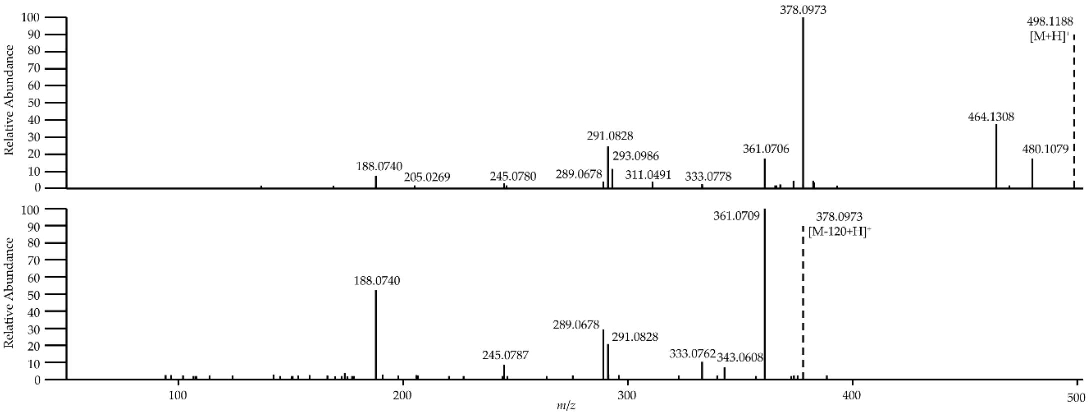

Figure S20. Fragmentation spectrum of the novel derivative **1**-B by the application of LC-MS/HRMS (LTQ Orbitrap XL™, CID, 35%). Spectra shown: MS² spectrum of the [M+H]⁺ ion (*m/z* 498.1188) and MS³ spectrum of the [M-120+H]⁺ ion (*m/z* 378.0973).

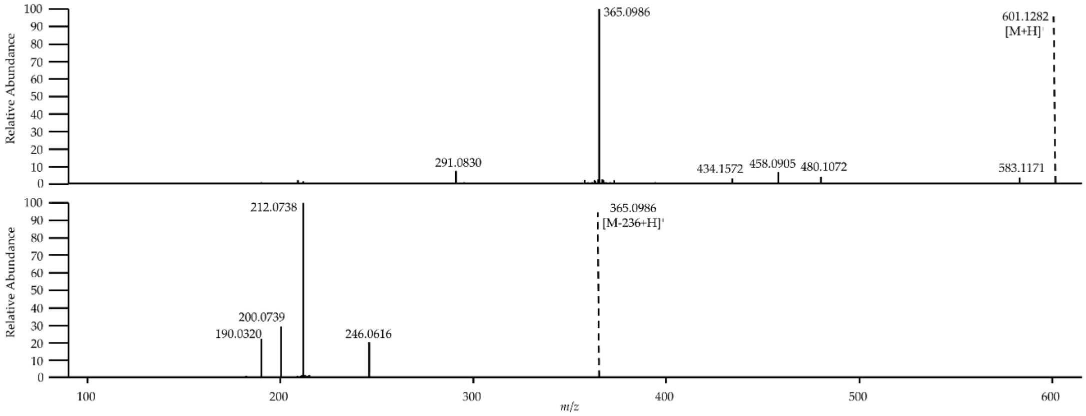

Figure S21. Fragmentation spectrum of the novel derivative **2**-B by the application of LC-MS/HRMS (LTQ Orbitrap XL™, CID, 35%). Spectra shown: MS^2 spectrum of the $[M+H]^+$ ion (*m/z* 601.1282) and MS^3 spectrum of the $[M-236+H]^+$ ion (*m/z* 365.0986).

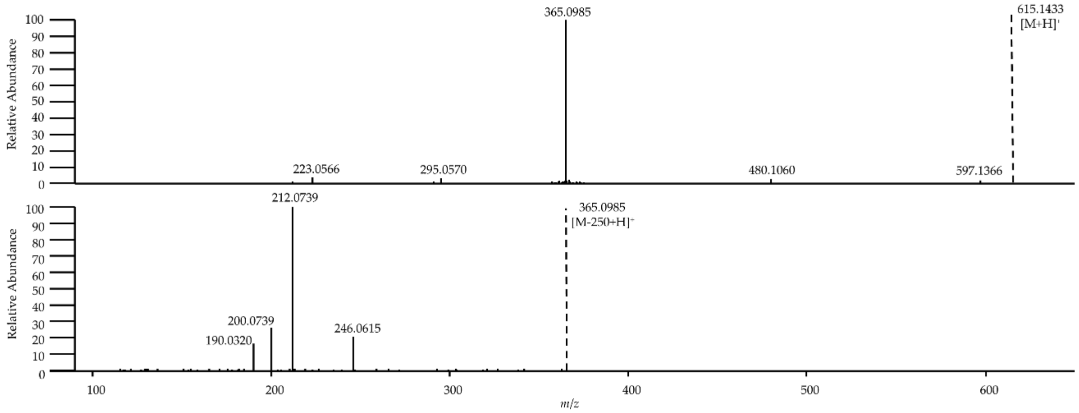

Figure S22. Fragmentation spectrum of the novel derivative **3**-B by the application of LC-MS/HRMS (LTQ Orbitrap XL™, CID, 35%). Spectra shown: MS^2 spectrum of the $[M+H]^+$ ion (*m/z* 615.1433) and MS^3 spectrum of the $[M-250+H]^+$ ion (*m/z* 365.0986).

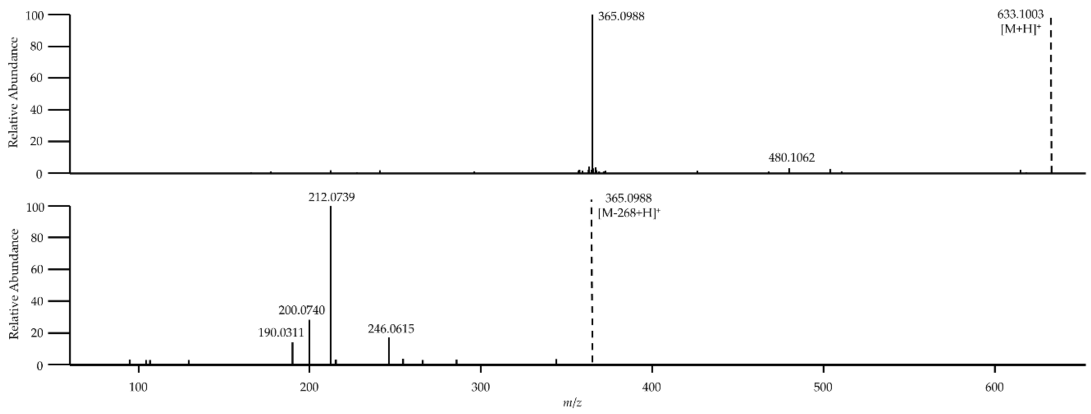

Figure S23. Fragmentation spectrum of the novel derivative **4**-B by the application of LC-MS/HRMS (LTQ Orbitrap XL™, CID, 35%). Spectra shown: MS^2 spectrum of the $[M+H]^+$ ion (*m/z* 633.1003) and MS^3 spectrum of the $[M-268+H]^+$ ion (*m/z* 365.0986).

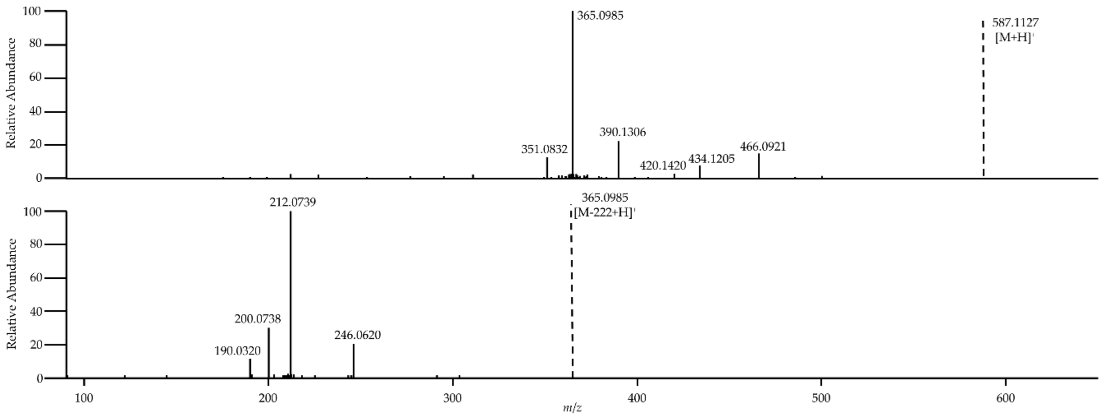

Figure S24. Fragmentation spectrum of the novel derivative **5**-B by the application of LC-MS/HRMS (LTQ Orbitrap XL™, CID, 35%). Spectra shown: MS^2 spectrum of the $[M+H]^+$ ion (*m/z* 587.1127) and MS^3 spectrum of the $[M-222+H]^+$ ion (*m/z* 365.0986).

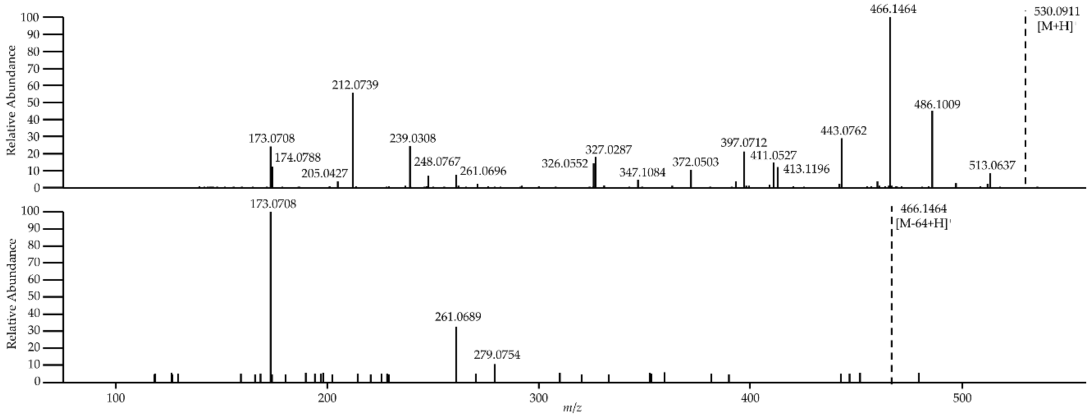

Figure S25. Fragmentation spectrum of the novel derivative **6**-B by the application of LC-MS/HRMS (LTQ Orbitrap XL™, CID, 35%). Spectra shown: MS^2 spectrum of the [M+H]$^+$ ion (*m/z* 530.0911) and MS^3 spectrum of the [M-64+H]$^+$ ion (*m/z* 466.1464).
